# Anxiety correlates with cortical surface area in subjective cognitive decline: APOE ε4 carriers versus APOE ε4 non-carriers

**DOI:** 10.1186/s13195-019-0505-0

**Published:** 2019-06-03

**Authors:** Yu Sun, Xiaoni Wang, Yinshan Wang, Haoming Dong, Jie Lu, Tohar Scheininger, Michael Ewers, Frank Jessen, Xi-Nian Zuo, Ying Han

**Affiliations:** 1 Department of Neurology, Xuanwu Hospital of Capital Medical University, Beijing, China; 2 CAS Key Laboratory of Behavioral Science and Research Center for Lifespan Development of Mind and Brain (CLIMB), Institute of Psychology, Beijing, China; 3 Department of Radiology, Xuanwu Hospital of Capital Medical University, Beijing, China; 4 Center for the Developing Brain, Child Mind Institute, New York, USA; 5 Institute for Stroke and Dementia Research (ISD), Ludwig-Maximilians-Universität (LMU), Munich, Germany; 6 Department of Psychiatry, Medical Faculty, University of Cologne, Cologne, Germany; 7 National Clinical Research Center for Geriatric Disorders, Beijing, China; 8 Beijing Institute of Geriatrics, Beijing, China; 9 Center of Alzheimer’s Disease, Beijing Institute for Brain Disorders, Beijing, China

**Keywords:** Subjective cognitive decline, Apolipoprotein E, Anxiety, Cortical morphometry, Alzheimer’s disease

## Abstract

**Background:**

Subjective cognitive decline (SCD) is characterized by self-reported cognitive deficits without measurable cognitive impairment. It has been suggested that individuals with SCD exhibited brain structural alterations in widespread cortical thinning or gray matter loss in the medial temporal and frontotemporal regions. Apolipoprotein E (*APOE*) ε4 allele is thought to be a genetic marker associated with risk of SCD. Neuropsychiatric symptoms may provide insight in detecting higher-risk elders for early Alzheimer’s disease as well. Therefore, we aim to explore the characteristics of brain morphology in SCD and to determine whether it is influenced by *APOE* ε4 as well as neuropsychiatric symptoms in SCD.

**Methods:**

A total of 138 cognitively normal older individuals from the SILCODE cohort underwent a clinical interview, neuropsychological assessments, a blood test, and MRI. A two-sample *t*-test was used to examine the cortex volume and bilateral cortical surface area alterations between SCD (*n* = 65) and controls (*n* = 73). A general linear model analysis was used to test for both main and interaction effects of clinical phenotype (SCD vs. controls) and *APOE* on global and regional cortex volume and bilateral cortical surface area and thickness. A multiple linear regression analysis was conducted to determine the effects of the *APOE* genotype on the relationships between morphometric features and neuropsychiatric symptoms in SCD.

**Results:**

Compared with controls, individuals with SCD showed decreased total cortical volumes and cortical surface area. SCD *APOE* ε4 carriers showed additive reduction in the right cortical surface area. The evaluation scores of anxiety symptoms were negatively associated with the right cortical surface area in SCD *APOE* 4 non-carriers.

**Conclusions:**

Individuals with SCD had an altered cortical surface area, and *APOE* genotype and anxiety symptoms are modified factors on the cortical surface area decrease in SCD.

**Trial registration:**

ClinicalTrials.gov (Identifier: NCT03370744). Registered 15 March 2017.

**Electronic supplementary material:**

The online version of this article (10.1186/s13195-019-0505-0) contains supplementary material, which is available to authorized users.

## Background

Subjective cognitive decline (SCD) is a clinical state characterized by subjective cognitive deficits without measurable cognitive impairment. Individuals with SCD may show a higher risk for biomarker patterns indicative of Alzheimer’s disease (AD) pathology, suggesting that SCD are at an increased risk for progressing to mild cognitive impairment (MCI) or AD [1–4]. Indeed, individuals with SCD constitute a heterogeneous population. To identify the specific characteristics of SCD that are associated with an increased likelihood of AD with affordable and easily accessible measures could help imply appropriate candidates for early detection in AD.

Previous studies have identified that individuals with SCD showed structural gray matter volume reductions and cortical thinning in the bilateral entorhinal cortex [5], medial temporal, and frontotemporal regions [6] compared to cognitively normal elders without cognitive complaints. While particular regions of the brain may be involved in the underlying pathology of AD, some abnormalities may also be present in a widespread form, thus producing global alterations to brain structure at a very early stage. SCD has known associations with an AD-like pattern of gray matter atrophy [7], and the widespread cortical thinning is associated with faster subsequent decline in memory [8]. Many studies have examined the brain volumetric and thickness measures in SCD; there is a scarcity of research investigating cortical surface area, an increasingly used brain morphology metric, which is ontogenetically and phylogenetically distinct from cortical thickness [9]. The cortical surface area is determined by symmetrical division of progenitor cells from the ventricular and subventricular zones of cortical layers, while the cortical thickness is formed by the asymmetrical division of radial glia [10]. Recent research has demonstrated that surface-based structure analysis may offer stronger statistical power than volume-based analysis in capturing subtle structural alterations as well as the effect of apolipoprotein E (*APOE*) genotype [11, 12]. Our previous work using combined resting-state functional and structural MR have found no gray matter differences in SCD compared to controls [13]. Thus, in this study, we employed surface-based analysis to detect cortical morphology which would be better suitable to manifest the subtle structural changes under early stages.

*APOE* ε4 allele is a well-established genetic risk factor for progression of sporadic AD, and influence of *APOE* genotype in SCD has aroused growing interests [14, 15]. Longitudinal studies have demonstrated that both memory complaints and *APOE* ε4 allele predict clinical cognitive decline in cognitively intact elderly individuals and additive effects were shown in individuals with both factors [16]. Recent meta-analysis indicated *APOE* ε4 was significantly associated with risk of having SCD in cognitively normal subjects as well as developing to AD in SCD [17]. Studies have found the significant interaction of SCD and *APOE* ε4, in which SCD *APOE* ε4 carriers performed worse on the episodic memory and showed smaller left hippocampal volumes [18], while other studies have not observed the differences associated with *APOE* ε4 statue in glucose metabolism and medial temporal lobe atrophy in SCD [19]. Thus, the *APOE* ε4 genetic effect on brain neurodegeneration as early as in SCD population remains ambiguous.

Another emerging line of research focusing on the association between neuropsychiatric symptoms (such as symptoms of depression and anxiety) and AD pathophysiologic abnormalities has suggested subtle neuropsychiatric symptoms as manifestations of AD progression [20] and higher risk for greater cognitive decline [21]. Previous studies have found depressive symptoms and higher trait neuroticism in SCD [22]. Longitudinal studies also have found that higher amyloid beta burden at baseline was associated with increasing anxious-depressive symptoms over time in cognitively normal older individuals [23], supporting that neuropsychiatric symptoms may provide insight in detecting participants at higher risk for preclinical AD. Cortical surface area is extensively used to detect brain structural alterations in psychiatric disorders [24, 25]. However, there is a lack of studies on whether and how neuropsychiatric symptoms are affecting the cortical surface area in SCD population.

For reasons above, we aim to investigate the characteristics of the cortical surface area in individuals with SCD compared to controls, and whether *APOE* ε4 statue and neuropsychiatric symptoms (e.g., depressive and/or anxiety) influenced the cortical surface area, which may help improve the sensitivity of structural imaging studies to provide a separate morphologic index of SCD with genetic and neuropsychiatric risk factors. We hypothesized that the cortical surface area would decrease in SCD compared with control subjects, and *APOE* ε4 allele may have additive effect on cortical reduction. We also estimated that subtle neuropsychiatric symptoms would be associated with cortical surface area alterations in SCD.

## Methods

### Participants

This study is part of the Sino-Longitudinal Cognitive Impairment and Dementia Study (SILCODE), which aimed to predict cognitive decline by utilizing neuroimaging techniques in the diagnosis and treatment of preclinical AD. SILCODE is a multicenter-based longitudinal observational study in China, mainly focusing on SCD, and also includes individuals with MCI, mild AD dementia, and control subjects. All subjects participated in a standardized clinical evaluation and physical examination, provided their medical history, had blood work taken, performed a battery of neuropsychological assessments, and received a structural MRI. Glucose metabolism and amyloid positron emission tomography were selectively conducted based on individual agreement.

The study was registered on ClinicalTrials.gov (Identifier: NCT03370744). The study protocol was approved by the institutional review board at Xuanwu Hospital in Capital Medical University, and all participants completed a written informed consent before taking part in study procedures.

Participants in the present study were recruited from March 20, 2017, to February 27, 2018, including 138 right-handed Han Chinese subjects (65 SCD and 73 controls) recruited from Xuanwu Hospital. A semi-structured interview used by the DELCODE study was employed for all participants to evaluate the details of self-reported cognitive decline [26]. The information about the onset time, concerns, comparison with others, and the history of visiting a physician not only memory domain but also language, attention, and executive were documented. Meanwhile, the informant reports in the evaluation of the self-reported information were also performed [27, 28]. SCD is defined with the following criteria [1]: (1) self-experienced persistent decline in memory rather than other domains of cognition within the last 5 years, (2) concerns related to SCD and a feeling of worsened performance when compared to others of the same age group as expressed to physicians via the structured interview, (3) cognitive decline confirmed by an another informant, and (4) performance on standardized neuropsychological tests within age-, gender-, and education-adjusted norms and failure to meet the criteria for MCI or dementia [29]. Individuals with no cognitive complaints or any concerns via the structured interview and normal performance on the standardized neuropsychological tests were included as controls.

Exclusion criteria are as follows: (1) current major psychiatric diagnoses such as severe depression and anxiety [e.g., Hamilton Depression Rating Scale (HAMD) > 24, Hamilton Anxiety Rating Scale (HAMA) > 29]. When mild and moderate symptoms of psychiatric diagnosis are suspected, patients will be not be excluded [30]. They will be evaluated by a psychiatrist to clear if the psychiatric diagnoses are the cause of SCD; (2) other neurological conditions which could cause cognitive decline (e.g., cerebrovascular disease, brain tumors, Parkinson’s disease, encephalitis, or epilepsy) rather than AD spectrum disorders; (3) other diseases which could cause cognitive decline (e.g., thyroid dysfunction, severe anemia, syphilis, or HIV); (4) history of psychosis or congenital mental growth retardation; (5) cognitive decline caused by traumatic brain injury; (6) those who could not complete the study protocol or with contraindications for MRI.

### Neuropsychological assessments

The neuropsychological test battery included tests that measure cognitive functioning in the domains of memory, language, and executive function. Auditory Verbal Learning Test-immediate recall (AVLT-IR), Auditory Verbal Learning Test-delayed recall (AVLT-DR), and Auditory Verbal Learning Test-recognition (AVLT-R) were administered to assess memory; Semantic Verbal Fluency Test (ANIMALS) and the Boston Naming Test (BNT) were administered to assess language; and Shape Trails Test Parts A and B were administered to assess executive function. In addition, all subjects were administered the Mini-Mental State Examination (MMSE), Montreal Cognitive Assessment Basic Version (MoCA-B) [31], Clinical Dementia Rating (CDR), Hamilton Depression Rating Scale (HAMD), Hamilton Anxiety Rating Scale (HAMA), and the Functional Activities Questionnaire (FAQ) to assess functioning across several different clinically relevant areas.

### *APOE* genotyping

DNA sequences for each subject were extracted for SNPs rs7412 and rs429358 from the *APOE* ε2/ε3/ε4 haplotype. *APOE* was genotyped using the standard Sanger sequencing method (Sangon, Shanghai, China) with the following primers: 5′-ACGCGGGCACGGCTGTCCAAGG-3′ (forward) and 5′-GGCGCTCGCGGATGGCGCTGA-3′ (reverse). *APOE* was amplified using the following conditions: 1 cycle of 98 °C for 10 s, 35 cycles of 72 °C for 5 s, 1 cycle of 72 °C for 5 min. PCR was performed in a final volume of 30 μl containing 10 pmol of forward and reverse primers, and 50 ng of genomic DNA template using PrimeSTAR HS DNA Polymerase with the GC Buffer (Takara Bio). In all, *APOE* genotype detection in 64 SCD and 70 controls were obtained. Due to the hemolysis of blood samples, we do not have the *APOE* genotype results of one SCD and three controls. When we considered the *APOE* effect in brain structure and behavior in SCD and controls, we excluded these four subjects.

### MR data acquisition

All participants were scanned on an integrated simultaneous 3.0 T TOF PET/MR (Signa PET/MR, GE Healthcare, WI, USA). 3D BRAVO T1-weighted sagittal images were obtained using the following parameters: repetition time/echo time = 6.9 ms/2.98 ms, flip angle = 12°, inversion time = 450 ms, field of view = 256 × 256 mm^2^, matrix = 256 × 256, slices = 192, slice thickness = 1 mm, no interslice gap, and voxel size = 1 × 1 × 1 mm^3^.

### Imaging analysis

For structural MR analyses, T1-weighted images were preprocessed by Connectome Computation System [32] (https://github.com/zuoxinian/CCS). This pipeline integrated multiple analysis platforms for processing multi-modal brain imaging data. Briefly, the whole workflow included as follows: (1) spatially adaptive non-local means de-noising, (2) rough inhomogeneity correction, (3) align image into MNI space, (4) inhomogeneity correction, (5) intensity normalization, (6) non-local intracranial cavity extraction [33], (7) gray matter/white matter segmentation, and (8) surface reconstruction. Steps from 1 to 6 were implemented in *volBrain* pipeline (http://volbrain.upv.es) [34]. The “recon-all” command in FreeSurfer (v6.0, https://surfer.nmr.mgh.harvard.edu) was used to run surface reconstruction. This pipeline has been previously described and well-validated to assess the cortical thickness (mm) and surface area (cm^2^) [35–37]. The entire procedure reconstructed individual surface models of white and gray matter surfaces and mapped brain morphometric measures of total cortical volume, and bilateral cortical surface area and cortical thickness onto these surface models [37].

In surface-based approaches, for each hemisphere, cortical thickness values were calculated as the shortest distance between the cortical gray/white boundary to the gray/CSF boundary, containing all FreeSurfer cortical regions of interest. The vertex-wise cortical surface area was calculated as the mean area of the associated triangular region. Total cortical volume is the product of thickness and surface area at each location across the cortical mantle.

Additionally, we also proceeded to calculate cortical volume values and bilateral surface area and thickness within a well-established large-scale network atlas containing default mode, dorsal and ventral attention, sensory motor, visual, fronto-parietal, and fronto-temporal, known as the seven-network parcellation proposed by Yeo et al. [38]. The annotation file of this parcellation on a fsaverage template was first resampled onto a subject native surface template to obtain the individualized the seven-network parcellation. Based on these individualized parcel information, the bilateral surface area as well as cortical thickness and cortical volume values over these networks was calculated by averaging all values inside the same network.

To detect the possible structural alterations in specific fronto-temporo-parietal cortices and gyri, the bilateral surface area and cortical thickness value in the frontal lobe, parietal lobe, temporal lobe, occipital lobe, cingulate, parahippocampal gyrus, and insula were calculated (see Additional file 1: Text S1 and Table S1-S7). Default mode network has been found to exhibit a breakdown of functional connectivity in AD even at an early stage. Thus, we calculated the surface area and cortical thickness value in regional components within the default mode, including bilateral posterior cingulated cortex (PCC), left prefrontal cortex (PFC), right medial PFC, right ventral PFC, parahippocampal cortex, bilateral temporal regions, and bilateral parietal regions to explore whether the cortical alterations have been found in SCD (see Additional file 1: Text S2 and Table S8-S12).

### Statistical analysis

SPSS (version 24.0, IBM) was utilized for statistical analyses. Group differences in demographic measures were tested using the independent samples *t*-test and the chi-square analyses for continuous and categorical variables, respectively. To compare cognitive variables, the two-way analysis of covariance (ANCOVA) was conducted with age, gender, and years of education as covariates. To compare the group differences in cortical morphometric features, independent samples *t*-test analyses in individuals with SCD and controls were conducted. For network-level morphometric measures, the ANCOVA was performed with intracranial volume as a covariate. To further explore whether the *APOE* genotype affects cortical morphometry in SCD, a two-way ANCOVA was performed to examine the existence of this interaction effect with phenotype (SCD vs. controls) and *APOE* genotype (ε4 carriers vs. non-carriers). The covariates for this analysis were age, gender, and years of education. Only findings with a two-tailed *P* < 0.05 (Bonferroni corrected) were reported. To determine the relationships between the cortical surface area and neuropsychiatric variables (HAMA and HAMD), a multiple linear regression analysis was conducted in the SCD group within the cortical surface area and total cortical volume. Age, gender, and education level were considered as covariates.

## Results

### Behavioral results

Table 1 summarizes the demographic characteristics and neuropsychological test results in all participants. The proportion of *APOE* ε4 carriers in the SCD and control groups were 25% and 20%, respectively. No significant differences were found in age, gender, education level, or *APOE* ε4 prevalence between the SCD and control subjects (all *P* > .1). There were significantly higher scores in the HAMA and HAMD (all *P* < .001) and poorer AVLT recognition (*P* = .005) performance in individuals with SCD, while discrepancies among other neuropsychological test scores were not significant.

**Table 1:** Subject demographics and neuropsychological assessments

	SCD (*n =* 65)	NC (*n =* 73)	*P* value
Age (years)	65.85 (4.85)	64.55 (5.52)	.147
Education	11.86 (2.70)	11.68 (3.31)	.734
Gender (male/female)	23/42	38/35	.138
*APOE* ε4 (+/−)^a^	16/48	14/56	.443
MMSE	28.65 (1.23)	28.79 (1.38)	.866
MoCA-B	25.25 (2.36)	25.79 (2.48)	.338
AVLT-IR	6.50 (1.13)	6.66 (1.68)	.432
AVLT-DR	6.57 (1.84)	6.95 (2.20)	.166
AVLT-R	21.95 (1.74)	22.56 (1.46)	.005
STT-A	63.49 (16.42)	64.56 (22.94)	.112
STT-B	143.85 (37.44)	139.22 (37.24)	.913
AFT	17.88 (4.28)	18.81 (4.60)	.232
BNT	24.74 (2.45)	25.26 (3.14)	.396
GDS	3.35 (3.16)	1.89 (1.80)	.382
HAMD	5.66 (4.26)	2.51 (2.64)	< .001
HAMA	6.34 (4.70)	2.65 (2.43)	< .001

*APOE* apolipoprotein E, *MMSE* Mini-Mental State Examination, *MOCA-B* Montreal Cognitive Assessment Basic Version, *AVLT-IR* Auditory Verbal Learning Test-immediate recall, *AVLT-DR* Auditory Verbal Learning Test-delay recall, *AVLT-R* Auditory Verbal Learning Test-recognition, *STT-A* Shape Trails Test Part A, *STT-B* Shape Trails Test Parts B, *AFT* semantic fluency (animals), *BNT* Boston Naming Test, *GDS* Geriatric Depression Scale, *HAMD* Hamilton Depression Rating Scale, *HAMA* Hamilton Anxiety Rating Scale

^a^*APOE* genotype results were included in SCD subjects (*N* = 64) and controls (*N* = 70)

### Between-group comparisons of cortical morphometric features

**Fig. 1:**
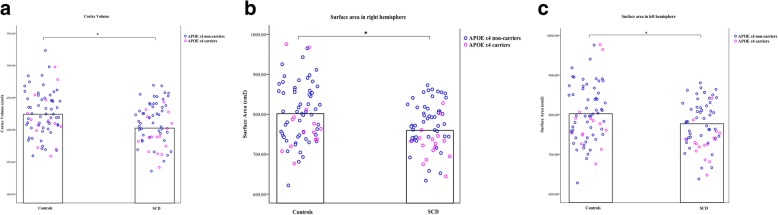
**a**–**c** Group differences in the cortex volume and bilateral surface area with a distribution of surface features in *APOE* ε4 carriers and non-carriers

Figure 1 and Table 2 show the significant differences in cortex volume and bilateral surface areas between the SCD and control groups (*P* < .05; Bonferroni corrected). Compared with the controls, individuals in the SCD group showed decreased total cortical volume, as well as decreased surface area in each hemisphere. There was no significant reduced cortical thickness in the individuals with SCD when compared to controls (Table 2). Furthermore, the SCD-related decrease in surface area seems to aggravate in *APOE* ε4 carriers (*P* = .086; Table 3; Fig. 1).

**Table 2:** Between-group differences in cortical morphometric features

	SCD	NC	*T*	*P* value
Cortex volume	408.9 ± 4.028	424.8 ± 4.094	2.748	.0068*
Surface area in the right hemisphere	766.4 ± 7.206	799.6 ± 8.711	2.943	.0038#*
Surface area in the left hemisphere	771 ± 7.316	801.3 ± 8.506	2.665	.0086*
Thickness in the right hemisphere	2.383 ± 0.009	2.383 ± 0.010	0.029	.9770
Thickness in the left hemisphere	2.379 ± 0.010	2.389 ± 0.010	0.688	.4927

Data is expressed as the means ± SEM; #*t*-test with Welch’s correction; *significant results with Bonferroni correction

**Table 3:** The estimated value of interaction effect between *APOE* and diagnosis in cortical morphometric features

	Groups	*APOE*	Estimation	95%CI	*P* value
Cortex volume	SCD	ε4 +	397.829 ± 8.076	381.848–413.810	.25
		ε4 −	416.300 ± 4.579	407.203–425.398	
	NC	ε4 +	420.606 ± 8.562	403.664–437.548	
		ε4 −	423.750 ± 4.312	415.217–432.283	
Surface area in right hemisphere	SCD	ε4 +	742.296 ± 13.860	714.871–769.722	.086
		ε4 −	782.810 ± 7.890	767.197–798.423	
	NC	ε4 +	794.782 ± 14.694	765.706–823.857	
		ε4 −	795.956 ± 7.400	781.312–810.600	
Surface area in left hemisphere	SCD	ε4 +	741.824 ± 15.268	711.613–772.036	.118
		ε4 −	786.760 ± 8.691	769.561–803.959	
	NC	ε4 +	795.066 ± 16.186	763.037–827.096	
		ε4 −	800.473 ± 8.152	784.342–816.605	
Thickness in right hemisphere	SCD	ε4 +	2.402 ± 0.020	2.362–2.442	.495
		ε4 −	2.381 ± 0.011	2.358–2.404	
	NC	ε4 +	2.378 ± 0.021	2.336–2.421	
		ε4 −	2.380 ± 0.011	2.359–2.401	
Thickness in left hemisphere	SCD	ε4 +	2.406 ± 0.021	2.365–2.447	.246
		ε4 −	2.374 ± 0.012	2.350–2.397	
	NC	ε4 +	2.381 ± 0.022	2.337–2.425	
		ε4 −	2.389 ± 0.011	2.367–2.411	

Data is expressed as the means ± SD; 95%CI, 95% confidence interval; covariates include age, gender, and year of education; ε4 + was *APOE* ε4 carriers while ε4 – was non-carriers

### Between-group comparisons of morphometric features on networks

There were no significant differences (Bonferroni corrected) of surface area and cortical volume in the visual, somatomotor, dorsal attention, ventral attention, limic, frontoparietal, and default networks between the SCD and control groups. The differences of cortical thickness and surface area in the frontal lobe, parietal lobe, temporal lobe, occipital lobe, cingulate, parahippocampal gyrus, and insula, as well as in bilateral posterior cingulated cortex (PCC), left prefrontal cortex (PFC), right medial PFC, right ventral PFC, parahippocampal cortex, bilateral temporal regions, and bilateral parietal regions within default mode, were not significant after Bonferroni correction.

### Relationship between cortical surface area and neuropsychological variables

**Fig. 2:**
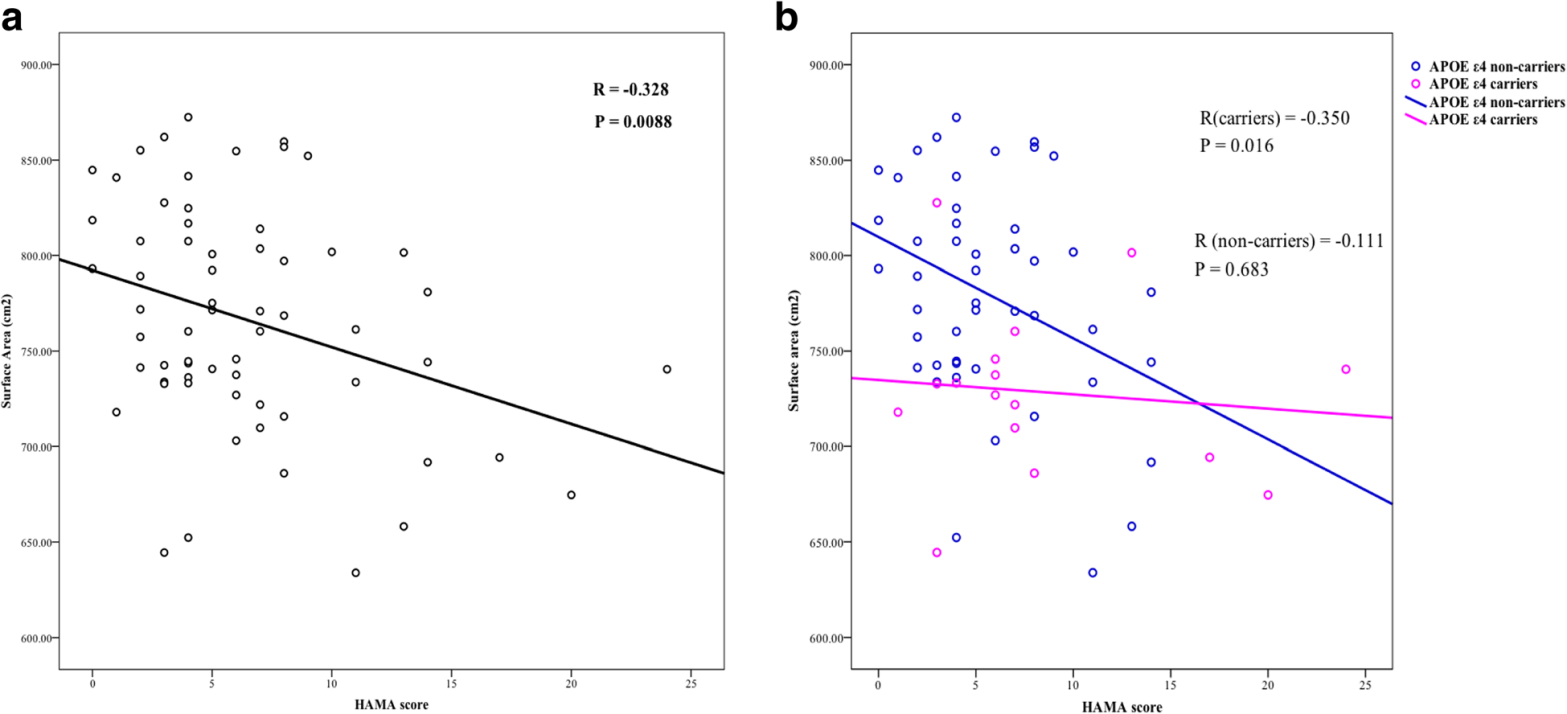
The relationship between the surface area and HAMA scores. **a** There is a significant negative correlation between the HAMA and surface area in the right hemisphere in SCD. **b** The negative correlation between the HAMA and surface area was significant in non-carriers in the SCD group

In the SCD group, there was a significant negative correlation between the HAMA and right hemisphere surface area (*r* = − 0.328, *P* = .0088; Fig. 2a). Moreover, the negative correlation between the HAMA and surface area was significant in non-carriers in the SCD group (*r* = − 0.350, *P* = .016; Fig. 2b). The correlation was reanalyzed after removing the two extreme HAMA score (> 20) values; however, the significant negative correlation between the HAMA and surface area in the right hemisphere remained essentially unchanged (*r* = − 0.289, *P* = .024). Both correlations between the HAMD and recognition scores and surface area were not significant. There was no significant correlation between the HAMA and surface area in controls (*r* = − 0.165, *P* = .181).

## Discussion

In line with previous literature [5, 39, 40], this study data indicated decreased cortical volume and surface area in the SCD group as compared to controls; however, there were no significant differences in structural alterations based on functional cortical networks. This suggests that the changes in global cortices in individuals with SCD were attributed to entire networks extensively, which may be related to intact cognitive performance. Cognitive complaints and *APOE* ε4 may have addictive effects on cortical surface area decline (*P* = .086; Table 3). Notably, anxiety scores were higher in SCD individuals and negatively correlated with surface area significantly in SCD *APOE* ε4 non-carriers.

Elevated anxiety and depression scores were found in the SCD group as compared to normal controls, which is similar with previous studies [21, 41]. It is possible that neuropsychiatric problems may act as an early risk factor for the decline in cognitive state, or alternatively, that neuropsychiatric problems perhaps be a prodromal symptom of upcoming cognitive impairment [21]. The Mayo Clinic Study of Aging conducted with a large sample of cognitively normal elders revealed that anxiety symptoms were associated with reduced insular thickness even after the result was adjusted for comorbid depressive symptoms, thus suggesting a structural alteration correlated to subclinical anxiety [42]. Consistent with the prior study [42], we found anxiety scores were negatively associated with the cortical surface area in SCD ε4 non-carriers, indicating anxiety could influence the structural changes gene-independently. One meta-analysis suggested there was no association between *APOE* carrierships or zygosity and neuropsychiatric symptoms including anxiety [43]. Previous research found that correlation between hippocampal volume reduction and late-life depression was not mediated by amyloid deposition [44], indicating neuropsychiatric symptoms may be associated with differences in pathway to brain morphemic alterations. This association which is not significant in SCD ε4 carriers verified our speculation that *APOE* ε4 and anxiety works on surface area distinctly and the relation between anxiety and cortical area reduction may be covered. Converging evidence showed a different atrophy pattern between *APOE* ε4 carriers and ε4 non-carriers [45, 46]. In young individuals (< 65 years old), *APOE* ε4 did not present detrimental effects and non-carriers showed severe cortical thinning [46]. In our study, SCD individuals were relatively young and association with anxiety may be eliminated. We cannot rule out the possibility that neuropsychiatric symptoms had less impact on the surface area in ε4 carriers due to some unrevealed factors. The lack of association in SCD ε4 carriers may also result from our sample size with ε4 carriers, so a large sample and longitudinal analyses are required. Our research indicates that neuropsychiatric problems play an important role in SCD, especially in *APOE* ε4 non-carriers. It may be beneficial to take subthreshold anxiety problems into consideration in SCD without *APOE* risk gene in clinical practice.

We found some enrichment of *APOE* ε4 in the SCD group (25%) as compared to the normal controls (21%), though it was not statistically significant between groups. In the study conducted by Zhang et al., the estimate for *APOE* ε4 carrier prevalence in SCD is 29% [47], which is similar to the results of this study. Further, a trend for the interaction between cognitive complaints and *APOE* genotype was found in predicting surface area reduction. Some previous research focusing on SCD and *APOE* ε4 genotype also showed a synergistic indicative effect for objective episodic memory decline [48] and hippocampal volume [18]. Though it is not significant, which may be due to the small sample, our results added to these works by suggesting the synergistic effect of SCD and *APOE* ε4.

It is interesting to mention the findings that both interaction between cognitive complaints and *APOE* genotype on surface area, and association between the HAMA and surface area were located in the right hemisphere. Donix et al. found *APOE* ε4 could modulate hemispheric asymmetry in cortical thickness, which is more significant in healthy controls. In AD patients, the asymmetry was less dependent on the *APOE* genotype [49]. So, one possible reason for the asymmetry interaction found in our study might be the differences in the effect of *APOE* ε4 on asymmetry between NC and SCD. The association between the HAMA and surface area found in our study is consistent with the “right hemisphere hypothesis” proposed by Gainotti [50, 51], which indicates a general dominance of the right hemisphere for all emotions. He found the hypothesis was supported by results from patients with frontotemporal lobar degeneration [52], but this assuming in AD patients still needs further study.

This data showed significant group difference in AVLT recognition between SCD and controls, while the performance of SCD was still within the age-adjusted normal range. The word recognition testing has been found sensitive to early memory impairments [53, 54] and progression to AD dementia in subjects with MCI [55]. The medial temporal lobes (MTL), especially the hippocampus, play an important role in successful memory retrieval [56]. Previous studies have showed that poorer recognition memory was associated with reduced MTL volume [57] and middle temporal gyrus connectivity [58]. Thus, whether the perceived memory decline in SCD is due to impaired encoding and consolidation of episodic information or a disruption in the retrieval of stored memory information remains a question worthy of future analysis.

In the current study, decreased cortical volume and surface area were found in the SCD group when compared to controls. Previous theories suggest that although cortical surface area and thickness were highly heritable, they were related to distinct genetic influences. The genetic influence on the surface area was explored to a greater degree, and early growth and development of the brain was found to be critical [59, 60]. The results of our study suggest that the cortical surface area rather than other metrics is influenced by genetic and emotional factors simultaneously, indicating that the surface area may be a more sensitive indicator for predicting AD. On the other hand, the discrepancy may be due to the differences in research standards and long continuous progress durations for individuals with SCD.

The present study presents some limitations and sheds an important light on the direction of future research. First, this is a cross-sectional data. The on-going multicenter longitudinal study, SILCODE, plays an important role in verifying the current assumptions and aims to establish a comprehensive estimation model for early detection as well as prediction in SCD. Second, with respect to interpretation of the correlation between the HAMA score and surface area in SCD ε4 carriers, the limited sample size should be taken into consideration. Third, in this study, we only used a HAMA single test to evaluate the anxiety symptoms for subjects with SCD. It would be important to include wider psychological tests to capture the neuropsychiatric performance in a more comprehensive manner.

## Conclusions

The current study focuses on the ability of cortical morphology in SCD individuals to interact with *APOE* genotype and anxiety thereby predicting cognitive decline, and hopes to improve the understanding of heterogeneity in SCD and enrich clinical trials on SCD. In conclusion, certain genetic and affective problems, namely *APOE* ε4 and subclinical anxiety symptoms, were identified as risk factors of early-stage AD and may modulate brain structural marker expressions in SCD.

## Additional file

Additional file 1: **Text S1.** Between-group comparisons of morphometric features in typical AD-related cortical regions. **Tables S1-S7.** Comparisons of bilateral surface area and cortical thickness in temporal lobe, parietal lobe, frontal lobe, occipital lobe, insula, cingulate and parahippocampal gyrus, separately, in SCD and controls. **Text S2.** Between-group comparisons of morphometric features in regions within default mode network (DMN). **Tables S8-S12.** Comparisons of surface area and cortical thickness value in bilateral parietal regions, bilateral posterior cingulated cortex (PCC), prefrontal cortex (PFC), bilateral temporal regions, and parahippocampal cortex within DMN, separately, in SCD and controls. (DOCX 331 kb)

## Data Availability

The datasets used and/or analyzed during the current study are available from the corresponding author on reasonable request.

## Abbreviations

AD: Alzheimer’s disease
*APOE*: Apolipoprotein E
AVLT-DR: Auditory Verbal Learning Test-delayed recall
AVLT-IR: Auditory Verbal Learning Test-immediate recall
AVLT-R: Auditory Verbal Learning Test-recognition
BNT: Boston Naming Test
CDR: Clinical Dementia Rating
FAQ: Functional Activities Questionnaire
HAMA: Hamilton Anxiety Rating Scale
HAMD: Hamilton Depression Rating Scale
MCI: Mild cognitive impairment
MMSE: Mini-Mental State Examination
MoCA-B: Montreal Cognitive Assessment Basic Version
SCD: Subjective cognitive decline
SILCODE: Sino-Longitudinal Cognitive Impairment and Dementia Study

## References

[1] Jessen F, Amariglio RE, van Boxtel M, Breteler M, Ceccaldi M, Chetelat G (2014). A conceptual framework for research on subjective cognitive decline in preclinical Alzheimer’s disease. Alzheimers Dement.

[2] Amariglio RE, Mormino EC, Pietras AC, Marshall GA, Vannini P, Johnson KA (2015). Subjective cognitive concerns, amyloid-beta, and neurodegeneration in clinically normal elderly. Neurology..

[3] Buckley RF, Hanseeuw B, Schultz AP, Vannini P, Aghjayan SL, Properzi MJ (2017). Region-specific Association of Subjective Cognitive Decline with Tauopathy Independent of global beta-amyloid burden. JAMA Neurol.

[4] Kielb S, Rogalski E, Weintraub S, Rademaker A (2017). Objective features of subjective cognitive decline in a United States national database. Alzheimers Dement.

[5] Jessen F, Feyen L, Freymann K, Tepest R, Maier W, Heun R (2006). Volume reduction of the entorhinal cortex in subjective memory impairment. Neurobiol Aging.

[6] Saykin AJ, Wishart HA, Rabin LA, Santulli RB, Flashman LA, West JD (2006). Older adults with cognitive complaints show brain atrophy similar to that of amnestic MCI. Neurology..

[7] Peter J, Scheef L, Abdulkadir A, Boecker H, Heneka M, Wagner M (2014). Gray matter atrophy pattern in elderly with subjective memory impairment. Alzheimers Dement.

[8] Verfaillie SCJ, Slot RE, Tijms BM, Bouwman F, Benedictus MR, Overbeek JM (2018). Thinner cortex in patients with subjective cognitive decline is associated with steeper decline of memory. Neurobiol Aging.

[9] Winkler AM, Greve DN, Bjuland KJ, Nichols TE, Sabuncu MR, Haberg AK (2018). Joint analysis of cortical area and thickness as a replacement for the analysis of the volume of the cerebral cortex. Cereb Cortex.

[10] Rakic P (1995). A small step for the cell, a giant leap for mankind: a hypothesis of neocortical expansion during evolution. Trends Neurosci.

[11] Dong Q, Zhang W, Wu J, Li B, Schron EH, McMahon T (2019). Applying surface-based hippocampal morphometry to study APOE-E4 allele dose effects in cognitively unimpaired subjects. Neuroimage Clin.

[12] Shi J, Lepore N, Gutman BA, Thompson PM, Baxter LC, Caselli RJ (2014). Genetic influence of apolipoprotein E4 genotype on hippocampal morphometry: an N = 725 surface-based Alzheimer’s disease neuroimaging initiative study. Hum Brain Mapp.

[13] Sun Y, Dai Z, Li Y, Sheng C, Li H, Wang X (2016). Subjective cognitive decline: mapping functional and structural brain changes-a combined resting-state functional and structural MR imaging study. Radiology..

[14] de Rojas I, Romero J, Rodriguez-Gomez O, Pesini P, Sanabria A, Perez-Cordon A (2018). Correlations between plasma and PET beta-amyloid levels in individuals with subjective cognitive decline: the Fundacio ACE healthy brain initiative (FACEHBI). Alzheimers Res Ther.

[15] Moreno-Grau S, Rodriguez-Gomez O, Sanabria A, Perez-Cordon A, Sanchez-Ruiz D, Abdelnour C (2018). Exploring APOE genotype effects on Alzheimer’s disease risk and amyloid beta burden in individuals with subjective cognitive decline: the FundacioACE healthy brain initiative (FACEHBI) study baseline results. Alzheimers Dement.

[16] Dik MG, Jonker C, Comijs HC, Bouter LM, Twisk JW, van Kamp GJ (2001). Memory complaints and APOE-epsilon4 accelerate cognitive decline in cognitively normal elderly. Neurology..

[17] Moreno-Grau S, Ruiz A (2016). Genome research in pre-dementia stages of Alzheimer’s disease. Expert Rev Mol Med.

[18] Striepens N, Scheef L, Wind A, Meiberth D, Popp J, Spottke A (2011). Interaction effects of subjective memory impairment and ApoE4 genotype on episodic memory and hippocampal volume. Psychol Med.

[19] Risacher SL, Kim S, Nho K, Foroud T, Shen L, Petersen RC (2015). APOE effect on Alzheimer’s disease biomarkers in older adults with significant memory concern. Alzheimers Dement.

[20] Ismail Z, Smith EE, Geda Y, Sultzer D, Brodaty H, Smith G (2016). Neuropsychiatric symptoms as early manifestations of emergent dementia: provisional diagnostic criteria for mild behavioral impairment. Alzheimers Dement.

[21] John A., Patel U., Rusted J., Richards M., Gaysina D. (2018). Affective problems and decline in cognitive state in older adults: a systematic review and meta-analysis. Psychological Medicine.

[22] Snitz BE, Lopez OL, McDade E, Becker JT, Cohen AD, Price JC (2015). Amyloid-beta imaging in older adults presenting to a memory clinic with subjective cognitive decline: a pilot study. J Alzheimers Dis.

[23] Donovan NJ, Locascio JJ, Marshall GA, Gatchel J, Hanseeuw BJ, Rentz DM (2018). Longitudinal association of amyloid beta and anxious-depressive symptoms in cognitively normal older adults. Am J Psychiatry.

[24] Bois C, Ronan L, Levita L, Whalley HC, Giles S, McIntosh AM (2015). Cortical surface area differentiates familial high risk individuals who go on to develop schizophrenia. Biol Psychiatry.

[25] Rus OG, Reess TJ, Wagner G, Zaudig M, Zimmer C, Koch K (2017). Structural alterations in patients with obsessive-compulsive disorder: a surface-based analysis of cortical volume, surface area and thickness. J Psychiatry Neurosci.

[26] Jessen F, Spottke A, Boecker H, Brosseron F, Buerger K, Catak C (2018). Design and first baseline data of the DZNE multicenter observational study on predementia Alzheimer’s disease (DELCODE). Alzheimers Res Ther.

[27] Molinuevo JL, Rabin LA, Amariglio R, Buckley R, Dubois B, Ellis KA (2017). Implementation of subjective cognitive decline criteria in research studies. Alzheimers Dement.

[28] Buckley R, Saling M, Ellis K, Rowe C, Maruff P, Macaulay LS (2015). Self and informant memory concerns align in healthy memory complainers and in early stages of mild cognitive impairment but separate with increasing cognitive impairment. Age Ageing.

[29] Bondi MW, Edmonds EC, Jak AJ, Clark LR, Delano-Wood L, McDonald CR (2014). Neuropsychological criteria for mild cognitive impairment improves diagnostic precision, biomarker associations, and progression rates. J Alzheimers Dis.

[30] Bhome R, Huntley JD, Price G, Howard RJ (2019). Clinical presentation and neuropsychological profiles of functional cognitive disorder patients with and without co-morbid depression. Cogn Neuropsychiatry.

[31] Chen KL, Xu Y, Chu AQ, Ding D, Liang XN, Nasreddine ZS (2016). Validation of the Chinese version of Montreal cognitive assessment basic for screening mild cognitive impairment. J Am Geriatr Soc.

[32] Xu T, Yang Z, Jiang L, Xing X-X, Zuo X-N (2015). A Connectome computation system for discovery science of brain. Sci Bull.

[33] Manjon JV, Eskildsen SF, Coupe P, Romero JE, Collins DL, Robles M (2014). Nonlocal intracranial cavity extraction. Int J Biomed Imaging.

[34] Manjon JV, Coupe P (2016). volBrain: An online MRI brain Volumetry system. Front Neuroinform.

[35] Dale AM, Fischl B, Sereno MI (1999). Cortical surface-based analysis. I. Segmentation and surface reconstruction. NeuroImage..

[36] Fischl B, Sereno MI, Dale AM (1999). Cortical surface-based analysis. II: inflation, flattening, and a surface-based coordinate system. NeuroImage..

[37] Fischl B, Dale AM (2000). Measuring the thickness of the human cerebral cortex from magnetic resonance images. Proc Natl Acad Sci U S A.

[38] Yeo BT, Krienen FM, Sepulcre J, Sabuncu MR, Lashkari D, Hollinshead M (2011). The organization of the human cerebral cortex estimated by intrinsic functional connectivity. J Neurophysiol.

[39] Fan LY, Lai YM, Chen TF, Hsu YC, Chen PY, Huang KZ (2018). Diminution of context association memory structure in subjects with subjective cognitive decline. Hum Brain Mapp.

[40] Meiberth D, Scheef L, Wolfsgruber S, Boecker H, Block W, Traber F (2015). Cortical thinning in individuals with subjective memory impairment. J Alzheimers Dis.

[41] Brigola AG, Manzini CSS, Oliveira GBS, Ottaviani AC, Sako MP, Vale FAC (2015). Subjective memory complaints associated with depression and cognitive impairment in the elderly: a systematic review. Dement Neuropsychol.

[42] Pink A, Przybelski SA, Krell-Roesch J, Stokin GB, Roberts RO, Mielke MM (2017). Cortical thickness and anxiety symptoms among cognitively normal elderly persons: the Mayo Clinic Study of Aging. J Neuropsychiatry Clin Neurosci.

[43] Banning LCP, Ramakers I, Deckers K, Verhey FRJ, Aalten P (2019). Apolipoprotein E and affective symptoms in mild cognitive impairment and Alzheimer’s disease dementia: a systematic review and meta-analysis. Neurosci Biobehav Rev.

[44] De Winter FL, Emsell L, Bouckaert F, Claes L, Jain S, Farrar G (2017). No association of lower hippocampal volume with Alzheimer’s disease pathology in late-life depression. Am J Psychiatry.

[45] Mattsson N, Ossenkoppele R, Smith R, Strandberg O, Ohlsson T, Jogi J (2018). Greater tau load and reduced cortical thickness in APOE epsilon4-negative Alzheimer’s disease: a cohort study. Alzheimers Res Ther.

[46] Kim J, Park S, Yoo H, Jang H, Kim Y, Kim KW (2018). The impact of APOE varepsilon4 in Alzheimer’s disease differs according to age. J Alzheimers Dis.

[47] Zhang T, Liu S, Zhang Y, Guan Y, Wang X, Zhao L (2017). Apolipoprotein E e4 allele is associated with subjective cognitive decline: a meta-analysis. Neuroepidemiology..

[48] Samieri C, Proust-Lima C, M MG, Okereke OI, Amariglio RE, Sperling RA (2014). Subjective cognitive concerns, episodic memory, and the APOE epsilon4 allele. Alzheimers Dement.

[49] Donix M, Burggren AC, Scharf M, Marschner K, Suthana NA, Siddarth P (2013). APOE associated hemispheric asymmetry of entorhinal cortical thickness in aging and Alzheimer’s disease. Psychiatry Res.

[50] Gainotti G (1972). Emotional behavior and hemispheric side of the lesion. Cortex..

[51] Gainotti Guido (2018). Emotions and the Right Hemisphere: Can New Data Clarify Old Models?. The Neuroscientist.

[52] Gainotti G (2019). The role of the right hemisphere in emotional and behavioral disorders of patients with frontotemporal lobar degeneration: an updated review. Front Aging Neurosci.

[53] Clark LR, Stricker NH, Libon DJ, Delano-Wood L, Salmon DP, Delis DC (2012). Yes/no versus forced-choice recognition memory in mild cognitive impairment and Alzheimer’s disease: patterns of impairment and associations with dementia severity. Clin Neuropsychol.

[54] Smirni D, Smirni P, Di Martino G, Fontana ML, Cipolotti L, Oliveri M (2019). Early detection of memory impairments in older adults: standardization of a short version of the verbal and nonverbal recognition memory test. Neurol Sci.

[55] De Simone MS, Perri R, Fadda L, Caltagirone C, Carlesimo GA (2019). Predicting progression to Alzheimer’s disease in subjects with amnestic mild cognitive impairment using performance on recall and recognition tests. J Neurol.

[56] Vaz AP, Inati SK, Brunel N, Zaghloul KA (2019). Coupled ripple oscillations between the medial temporal lobe and neocortex retrieve human memory. Science..

[57] Bennett IJ, Stark SM, Stark CEL. Recognition memory dysfunction relates to hippocampal subfield volume: a study of cognitively Normal and mildly impaired older adults. J Gerontol B Psychol Sci Soc Sci. 2018. 10.1093/geronb/gbx181. 10.1093/geronb/gbx181 PMC6748802 29401233

[58] Matura S, Prvulovic D, Butz M, Hartmann D, Sepanski B, Linnemann K (2014). Recognition memory is associated with altered resting-state functional connectivity in people at genetic risk for Alzheimer’s disease. Eur J Neurosci.

[59] Panizzon MS, Fennema-Notestine C, Eyler LT, Jernigan TL, Prom-Wormley E, Neale M (2009). Distinct genetic influences on cortical surface area and cortical thickness. Cereb Cortex.

[60] Jha SC, Xia K, Schmitt JE, Ahn M, Girault JB, Murphy VA (2018). Genetic influences on neonatal cortical thickness and surface area. Hum Brain Mapp.

