# Supplementary material for: Anxiety correlates with cortical surface area in subjective cognitive decline: APOE ε4 carriers versus APOE ε4 non-carriers
Source: Alzheimers Res Ther. 2019 Jun 3;11:50. doi: 10.1186/s13195-019-0505-0 (PMC6547570; doi:10.1186/s13195-019-0505-0)
Supplement: Supplementary file 1 — Text S1. Between-group comparisons of morphometric features in typical AD-related cortical regions. Tables S1-S7. Comparisons of bilateral surface area and cortical thickness in temporal lobe, parietal lobe, frontal lobe, occipital lobe, insula, cingulate and parahippocampal gyrus, separately, in SCD and controls. Text S2. Between-group comparisons of morphometric features in regions within default mode network (DMN). Tables S8-S12. Comparisons of surface area and cortical thickness value in bilateral parietal regions, bilateral posterior cingulated cortex (PCC), prefrontal cortex (PFC), bilateral temporal regions, and parahippocampal cortex within DMN, separately, in SCD and controls. (DOCX 331 kb) [file 13195_2019_505_MOESM1_ESM.docx]

**Additional file 1**

**Text S1. Between-group comparisons of morphometric features in typical AD-related cortical regions**

To detect the possible structural alterations in specific fronto-temporo-parietal cortices and gyri, bilateral surface area and cortical thickness value in frontal lobe, parietal lobe, temporal lobe, occipital lobe, cingulate, parahippocampal gyrus and insula were calculated. The annotation file of this parcellation on fsaverage template was first resample onto subject native surface template to obtain the individualized defined parcellation. Based on these individualized parcels information bilateral surface area as well as cortical thickness and cortical volume values over these regions were calculated by average all values inside the same network. The ANCOVA was performed to examine group differences in SCD and Controls with age, gender, years of education and intracranial volume as a covariate. The results showed no significant differences among these defined regions (Table S1-7; Bonferroni corrected).

Table S1

| **Temporal lobe** | **Groups** | Estimation | 95% IC | **P value** |
| --- | --- | --- | --- | --- |
| Surface area in left hemisphere | SCD | 109.974±0.956 | 108.081 – 111.868 | 0.232 |
|  | NC | 111.705±1.070 | 109.585 – 113.824 |  |
| Surface area in right hemisphere | SCD | 106.670±0.962 | 104.764 – 108.576 | 0.053 |
|  | NC | 109.506±1.077 | 107.372 – 111.641 |  |
| Thickness in left hemisphere | SCD | 33.825±0.346 | 33.139 – 34.511 | 0.201 |
|  | NC | 34.495 ±0.388 | 33.727 – 35.263 |  |
| Thickness in right hemisphere | SCD | 32.916±0.347 | 32.229 – 33.603 | 0.219 |
|  | NC | 33.562±0.388 | 32.793 – 34.332 |  |

Table S2

| **Parietal lobe** | **Groups** | Estimation | 95% IC | **P value** |
| --- | --- | --- | --- | --- |
| Surface area in left hemisphere | SCD | 203.075±2.052 | 199.010 – 207.141 | 0.180 |
|  | NC | 207.245±2.298 | 202.693 – 211.797 |  |
| Surface area in right hemisphere | SCD | 202.567±2.218 | 198.172 – 206.962 | 0.027 |
|  | NC | 210.036±2.484 | 205.115 – 214.956 |  |
| Thickness in left hemisphere | SCD | 49.605±0.607 | 48.403 – 50.806 | 0.526 |
|  | NC | 50.185 ±0.679 | 48.840 – 51.531 |  |
| Thickness in right hemisphere | SCD | 49.088±0.602 | 47.895 – 50.281 | 0.118 |
|  | NC | 50.517±0.674 | 49.182 – 51.853 |  |

Table S3

| **Frontal lobe** | **Groups** | Estimation | 95% IC | **P value** |
| --- | --- | --- | --- | --- |
| Surface area in left hemisphere | SCD | 264.305±2.310 | 259.728 – 268.882 | 0.077 |
|  | NC | 270.518±2.587 | 265.394 – 275.643 |  |
| Surface area in right hemisphere | SCD | 266.120±2.302 | 261.560 – 270.679 | 0.387 |
|  | NC | 269.126±2.577 | 264.021 – 274.232 |  |
| Thickness in left hemisphere | SCD | 73.931±0.723 | 72.499 – 75.361 | 0.646 |
|  | NC | 74.433 ±0.810 | 72.829 – 76.037 |  |
| Thickness in right hemisphere | SCD | 73.794±0.682 | 72.442 – 75.146 | 0.792 |
|  | NC | 73.523±0.764 | 72.009 – 75.036 |  |

Table S4

| **Occipital lobe** | **Groups** | Estimation | 95% IC | **P value** |
| --- | --- | --- | --- | --- |
| Surface area in left hemisphere | SCD | 101.608±1.510 | 98.617 – 104.599 | 0.173 |
|  | NC | 104.727±1.691 | 101.378 – 108.076 |  |
| Surface area in right hemisphere | SCD | 103.723±1.410 | 100.929 – 106.518 | 0.074 |
|  | NC | 107.547±1.579 | 104.418 – 110.675 |  |
| Thickness in left hemisphere | SCD | 20.149±0.319 | 19.517 – 20.782 | 0.103 |
|  | NC | 20.939 ±0.357 | 20.232 – 21.647 |  |
| Thickness in right hemisphere | SCD | 20.946±0.331 | 20.291 – 21.601 | 0.095 |
|  | NC | 21.784±0.370 | 21.050 – 22.517 |  |

Table S5

| **Insula** | **Groups** | Estimation | 95% IC | **P value** |
| --- | --- | --- | --- | --- |
| Surface area in left hemisphere | SCD | 22.667±0.284 | 22.105 – 23.229 | 0.008 |
|  | NC | 23.829±0.317 | 23.200 – 24.458 |  |
| Surface area in right hemisphere | SCD | 22.634±0.306 | 22.029 – 23.239 | 0.878 |
|  | NC | 22.704±0.342 | 22.027 – 23.382 |  |
| Thickness in left hemisphere | SCD | 6.570±0.089 | 6.393 – 6.746 | 0.144 |
|  | NC | 6.767 ±0.100 | 6.569 – 6.694 |  |
| Thickness in right hemisphere | SCD | 6.575±0.090 | 6.396 – 6.754 | 0.793 |
|  | NC | 6.611±0.101 | 6.410 – 6.812 |  |

Table S6

| **Cingulate** | **Groups** | Estimation | 95% IC | **P value** |
| --- | --- | --- | --- | --- |
| Surface area in left hemisphere | SCD | 31.238±0.396 | 30.454 – 32.022 | 0.044 |
|  | NC | 32.449±0.443 | 31.571 – 33.327 |  |
| Surface area in right hemisphere | SCD | 28.949±0.425 | 28.107 – 29.790 | 0.067 |
|  | NC | 30.418±0.476 | 29.476 – 31.360 |  |
| Thickness in left hemisphere | SCD | 8.430±0.115 | 8.203 – 8.657 | 0.023 |
|  | NC | 8.748 ±0.128 | 8.494 – 9.002 |  |
| Thickness in right hemisphere | SCD | 8.194±0.137 | 7.924 – 8.465 | 0.119 |
|  | NC | 8.518±0.153 | 8.215 – 8.821 |  |

Table S7

| **Parahippocampal gyrus** | **Groups** | Estimation | 95% IC | **P value** |
| --- | --- | --- | --- | --- |
| Surface area in left hemisphere | SCD | 43.401±0.463 | 42.483 – 44.318 | 0.100 |
|  | NC | 44.556±0.519 | 43.528 – 45.583 |  |
| Surface area in right hemisphere | SCD | 42.391±0.494 | 41.413 – 43.369 | 0.341 |
|  | NC | 43.102±0.553 | 42.007 – 44.197 |  |
| Thickness in left hemisphere | SCD | 14.867±0.193 | 14.484 – 15.249 | 0.114 |
|  | NC | 15.330 ±0.216 | 14.902 – 15.756 |  |
| Thickness in right hemisphere | SCD | 14.740±0.180 | 14.384 – 15.097 | 0.463 |
|  | NC | 14.940±0.201 | 14.541 – 15.339 |  |

**Text S2. Between-group comparisons of morphometric features in regions within default mode network**

Default mode network has been found to exhibit a breakdown of functional connectivity in AD even at early stage. Thus, we calculated surface area and cortical thickness value in regional components within DMN, including bilateral posterior cingulated cortex (PCC), left prefrontal cortex (PFC), right medial PFC, right ventral PFC, parahippocampal cortex, bilateral temporal regions, bilateral parietal regions to explore whether the cortical alterations have been found in SCD. The annotation file of this parcellation on fsaverage template was first resample onto subject native surface template to obtain the individualized defined parcellation. Based on these individualized parcels information bilateral surface area as well as cortical thickness and cortical volume values over these regions were calculated by average all values inside the same network. The ANCOVA was performed to examine group differences in SCD and Controls with age, gender, years of education and intracranial volume as a covariate. The results showed no significant differences among these defined regions (Table S8-12; Bonferroni corrected).

Table S8

| **Parietal regions in DMN** | **Groups** | Estimation | 95% IC | **P value** |
| --- | --- | --- | --- | --- |
| Surface area in left hemisphere | SCD | 22.747±0.401 | 21.954 – 23.541 | 0.585 |
|  | NC | 23.078±0.448 | 22.189 – 23.966 |  |
| Surface area in right hemisphere | SCD | 15.277±0.410 | 14.465 – 16.090 | 0.609 |
|  | NC | 15.594±0.459 | 14.685 – 16.504 |  |
| Thickness in left hemisphere | SCD | 6.195±0.111 | 5.975 – 6.415 | 0.678 |
|  | NC | 6.265 ±0.124 | 6.018 – 6.511 |  |
| Thickness in right hemisphere | SCD | 4.040±0.122 | 3.799 – 4.282 | 0.862 |
|  | NC | 4.072±0.136 | 3.802 – 4.342 |  |

Table S9

| **PCC in DMN** | **Groups** | Estimation | 95% IC | **P value** |
| --- | --- | --- | --- | --- |
| Surface area in left hemisphere | SCD | 20.729±0.299 | 19.700 – 20.886 | 0.019 |
|  | NC | 21.364±0.335 | 20.700 – 22.028 |  |
| Surface area in right hemisphere | SCD | 15.422±0.258 | 14.912 – 15.932 | 0.067 |
|  | NC | 16.140±0.288 | 15.568 – 16.711 |  |
| Thickness in left hemisphere | SCD | 5.837±0.076 | 5.687 – 5.987 | 0.410 |
|  | NC | 5.931 ±0.085 | 5.764 – 6.099 |  |
| Thickness in right hemisphere | SCD | 4.478±0.072 | 4.336 – 4.620 | 0.152 |
|  | NC | 4.634±0.080 | 4.475 – 4.793 |  |

Table S10

| **PFC in DMN** | **Groups** | Estimation | 95% IC | **P value** |
| --- | --- | --- | --- | --- |
| Surface area in left hemisphere | SCD | 69.333±0.744 | 67.859 – 70.806 | 0.043 |
|  | NC | 71.626±0.833 | 69.977 – 73.275 |  |
| Thickness in left hemisphere | SCD | 21.068±0.251 | 20.571 – 21.564 | 0.459 |
|  | NC | 21.348±0.281 | 20.792 – 21.904 |  |
| Medial PFC Surface area in right hemisphere | SCD | 43.036±0.528 | 41.971 – 44.101 | 0.215 |
|  | NC | 44.045 ±0.602 | 42.853 – 45.238 |  |
| Medial PFC Thickness in right hemisphere | SCD | 12.768±0.181 | 12.409 – 13.127 | 0.903 |
|  | NC | 12.801±0.203 | 12.399– 13.203 |  |
| Ventral PFC Surface area in right hemisphere | SCD | 5.502±0.082 | 5.339 – 5.665 | 0.966 |
|  | NC | 5.507±0.092 | 5.324 – 5.690 |  |
| Ventral PFC Thickness in right hemisphere | SCD | 1.864±0.031 | 1.803 – 1.925 | 0.889 |
|  | NC | 1.857±0.035 | 1.789 – 1.926 |  |

Table S11

| **Temporal regions in DMN** | **Groups** | Estimation | 95% IC | **P value** |
| --- | --- | --- | --- | --- |
| Surface area in left hemisphere | SCD | 32.951±0.371 | 32.217 – 33.685 | 0.559 |
|  | NC | 33.277±0.415 | 32.455 – 34.099 |  |
| Surface area in right hemisphere | SCD | 23.966±0.321 | 23.331 – 24.601 | 0.276 |
|  | NC | 24.494±0.359 | 23.783 – 25.204 |  |
| Thickness in left hemisphere | SCD | 9.872±0.124 | 9.626 – 10.117 | 0.494 |
|  | NC | 10.000 ±0.139 | 9.725 – 10.275 |  |
| Thickness in right hemisphere | SCD | 7.310±0.113 | 7.085 – 7.534 | 0.499 |
|  | NC | 7.425±0.127 | 7.174 – 7.677 |  |

Table S12

| **Parahippocampal in DMN** | **Groups** | Estimation | 95% IC | **P value** |
| --- | --- | --- | --- | --- |
| Surface area in left hemisphere | SCD | 1.272±0.021 | 1.229 – 1.314 | 0.846 |
|  | NC | 1.265±0.024 | 1.218 – 1.313 |  |
| Thickness in left hemisphere | SCD | 0.379±0.010 | 0.359 – 0.399 | 0.847 |
|  | NC | 0.376±0.011 | 0.353 – 0.398 |  |

A.


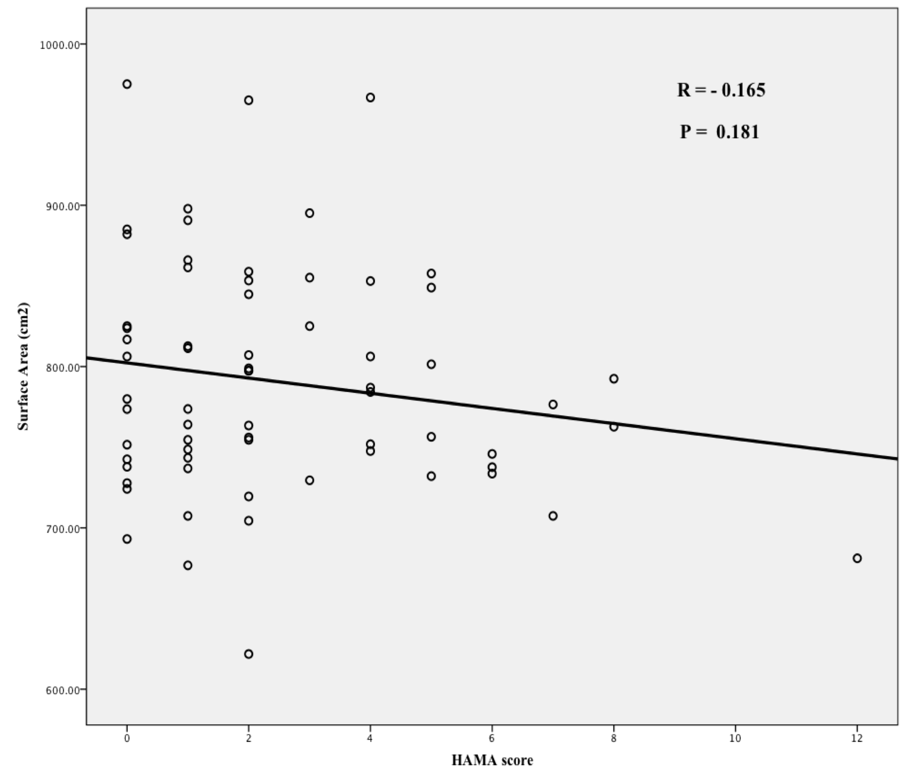


B.


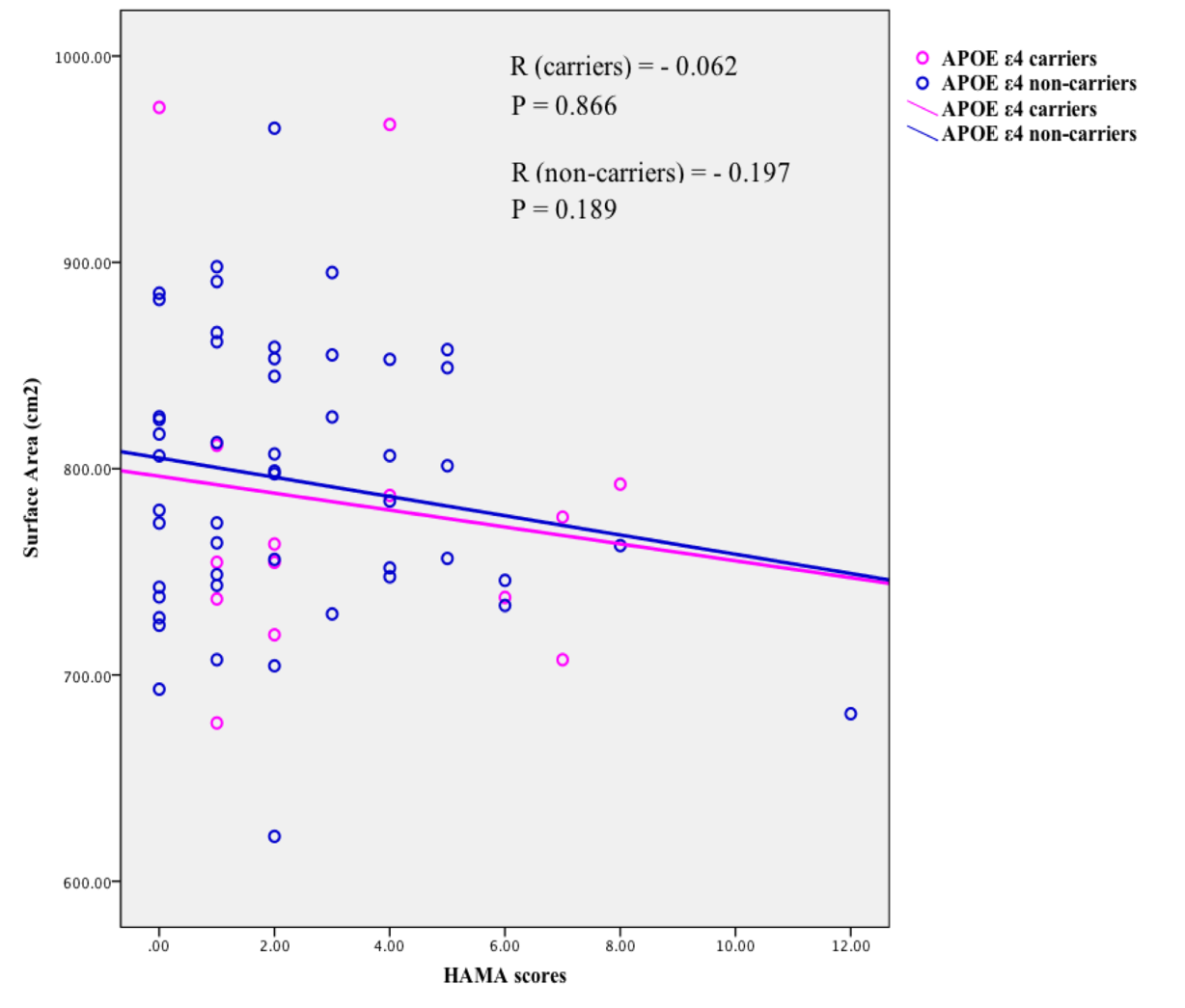


**Figure S1.** The relationship between surface area and HAMA scores in Controls. (A) There is no significant correlation between HAMA and surface area in right hemisphere in controls; (B) The correlations between HAMA and surface area in APOE ε4 carriers and non-carriers, separately.


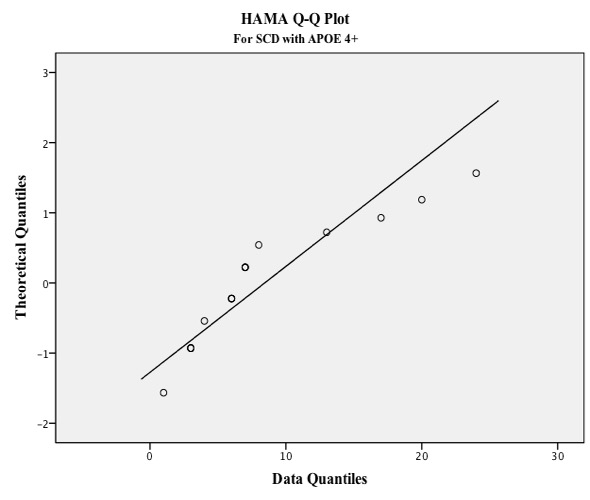


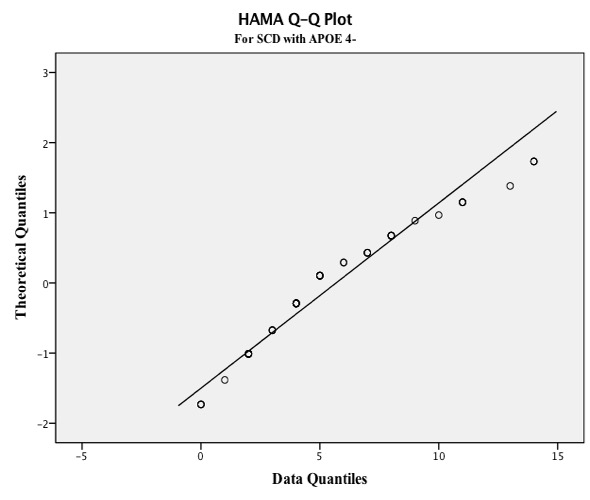


**Figure S2.** The P-P plot is to test the normality of HAMA scores in SCD APOE ε4 carriers and non-carriers, separately.
